# Comparative genomic analysis of *Staphylococcus* spp. and *Mammaliicoccus sciuri* suggests potential sharing between dogs and their owners

**DOI:** 10.3389/fpubh.2026.1879619

**Published:** 2026-10-02

**Authors:** Fernanda Cruz Bonnard, Luciana Guimarães, Alessandra Beatriz Santos Rondon Souza, Felipe Ramos Pinheiro, Isabella Thomaz da Silva, Izabel Mello Teixeira, Felipe Piedade Gonçalves Neves, Eliane de Oliveira Ferreira, Maria Isabel Di Azevedo, Bruno Penna

**Affiliations:** 1 Laboratório de Cocos Gram-positivos, Instituto Biomédico, Universidade Federal Fluminense (UFF), Niterói, Brazil; 2 Departamento Microbiologia Médica, IMPPG, UFRJ, Rio de Janeiro, Brazil

**Keywords:** antimicrobial resistance, companion animals, one health, *Staphylococcus* spp., whole-genome sequencing

## Abstract

**Introduction:**

Staphylococci are a diverse group of bacteria commonly found on the skin and mucosal surfaces of humans and animals. Members of the genus can transition from commensalism to opportunistic pathogenicity. The extent to which close contact facilitates transmission of staphylococci remains misunderstood, particularly at the genomic level. In this context, the present study aimed to investigate the possible transmission of *Staphylococcus* spp. and *Mammaliicoccus sciuri* between dogs and their owners.

**Materials and methods:**

For this, we used a comparative genomics framework to investigate genomic relatedness, antimicrobial resistance, and virulence gene profiles. A total of 27 bacterial isolates were analyzed by whole-genome sequencing, obtained from thirteen dog–owner pairs selected from a previous study involving 50 dogs and 34 owners, in which the same Staphylococcus spp. was identified in both hosts. The isolates were initially identified by matrix-assisted laser desorption/ionization time-of-flight mass spectrometry (MALDI-TOF), followed by analyses of resistance and virulence genes, molecular typing, and genomic similarity.

**Results and discussion:**

The resistome analysis revealed the presence of clinically relevant antimicrobial resistance determinants. Virulence profiling identified genes associated with host colonization and persistence. Average Nucleotide Identity (ANI) revealed high levels of similarity in several dog– owner pairs (*n* = 9 pairs, 64.3%), with values exceeding 99.7% and broad genomic coverage, indicating a high degree of genomic relatedness and potential strain sharing. Genomic distance analyses and core-genome phylogeny corroborated these findings, revealing both highly related pairs and genetically distinct lineages. Notably, some pairs exhibited highly similar genomes across multiple analytical approaches, consistent with close genomic relatedness and possible epidemiological linkage, whereas others demonstrated substantial genetic divergence despite close contact. Taken together, the results suggest that bacterial sharing between dogs and their owners may occur in a heterogeneous manner, involving both potential strain sharing and the independent circulation of different lineages. This dual pattern highlights the complexity of microbial exchange within household environments and underscores the role of companion animals as potential reservoirs of antimicrobial-resistant bacteria. These findings reinforce the importance of One Health–based approaches for understanding the circulation and genomic relatedness of staphylococci at the human–animal interface and provide genomic evidence to support surveillance efforts involving companion animals and their owners.

## Introduction

The increasingly close interaction between companion animals and their owners has expanded opportunities for interspecies microbial exchange, particularly in shared household environments. In recent decades, changes in human–animal relationships, including increased indoor cohabitation and closer physical contact, have intensified this interface, creating conditions conducive to interspecies microbial transmission (1). This close relationship facilitates the transmission of skin-associated bacteria from animals, whether symptomatic or asymptomatic, to their owners, especially among immunocompromised individuals, and also represents an occupational risk for professionals such as veterinarians (2). Additionally, both livestock and companion animals have been implicated as important contributors to the dissemination of resistant bacteria within a complex and interconnected One Health framework for the emergence and spread of antimicrobial resistance (AMR) (3).

In this scenario, bacteria of the genus *Staphylococcus* spp. stand out due to their widespread distribution in the skin and mucosal microbiota of humans and animals, as well as their pathogenic potential. These organisms comprise a diverse group of commensal and opportunistic pathogens that can transition between colonization and infection depending on host and environmental factors (4). Species within this genus are frequently associated with opportunistic infections and exhibit a high capacity to acquire and disseminate antimicrobial resistance genes, which limits available therapeutic options (5). This adaptability is largely driven by the acquisition of mobile genetic elements and horizontal gene transfer, facilitating rapid genomic diversification (6).

Similarly, *Mammaliicoccus sciuri*, formerly classified within the genus *Staphylococcus*, has also been recognized as a reservoir of resistance genes. *M. sciuri* is also able to adapt to different ecological niches, including animals, humans, and environmental sources, contributing to the dissemination dynamics of these genetic determinants (7). Importantly, this species has been increasingly implicated as a potential donor of resistance genes to more clinically relevant staphylococcal species, highlighting its role in the evolution of antimicrobial resistance (8, 9).

The zoonotic transmission of methicillin-resistant Staphylococci has been considered a relevant and potentially unavoidable phenomenon, representing a challenge for both veterinary medicine and public health (5). Close contact within households, including shared spaces and frequent physical interaction, has been identified as a key driver of bidirectional transmission between humans and companion animals (10–12). This scenario reinforces the importance of One Health–based approaches for understanding the circulation of antimicrobial-resistant microorganisms at the human–animal interface and highlights the need for integrated surveillance strategies involving both companion animals and their owners (13).

Genomic approaches—particularly whole-genome sequencing—have enabled a more detailed characterization of resistance and virulence factors, as well as the evolutionary relationships among bacterial strains. Comparative genomics studies have been increasingly used to elucidate phylogenetic relationships and identify dissemination patterns among *Staphylococcus* spp. isolates (14–16). Comparative genomic approaches enable the integration of genomic similarity metrics, phylogenetic inference, and gene content analyses, providing useful frameworks for investigating genomic relatedness and potential bacterial sharing at the human–animal interface (17). However, important gaps remain in understanding the relationships among these genetic profiles, the epidemiology of infections, and host–pathogen interactions (18), particularly in the context of household-level transmission involving companion animals. We hypothesized that dogs and their owners harbor genetically related *Staphylococcus* spp. and *Mammaliicoccus sciuri* strains, reflecting potential bacterial sharing within household environments. To test this hypothesis, the present study aimed to investigate the possible transmission of *Staphylococcus* spp. and *Mammaliicoccus sciuri* between dogs and their owners by identifying clones and conducting comparative genome analysis. Additionally, resistance genes and virulence factors were evaluated to better characterize the genomic features of these microorganisms in household environments, providing insights into potential epidemiological links, bacterial diversity, and antimicrobial resistance patterns at the human–animal interface from a One Health perspective.

## Materials and methods

### Characterization of isolates

Previously identified isolates belonging to several *Staphylococcus* species and *Mammaliicoccus sciuri* were analyzed All isolates are from the collection of the Gram-Positive Cocci Laboratory at Fluminense Federal University. The study was approved by the human research ethics committee (CAAE: 14348319.4.0000.5243) and the animal use ethics committee (CEUA No. 1008), with informed consent obtained from all participants.

The isolates were originally characterized in a previous study (19), in which samples from 50 dogs and 34 owners were analyzed. Bacterial identification was performed using matrix-assisted laser desorption/ionization time-of-flight mass spectrometry (MALDI-TOF), followed by detection of the *mecA* gene by PCR and evaluation of antimicrobial susceptibility profiles by disk diffusion, in accordance with the recommendations of the Clinical and Laboratory Standards Institute (20). The microbiological and antimicrobial susceptibility results from the original study are summarized in Table 1.

**Table 1:** Characterization of bacterial isolates recovered from dog–owner pairs, including host origin, isolate identification, bacterial species, phenotypic antimicrobial resistance profile, and detection of the *mecA* gene (by PCR).

Pair	Host	ID sample	Bacterial species	Antimicrobial susceptibility profile	*mecA* Gene
1	Dog	SW_dog_07	*S. warneri*	cfo, pen, eri, cli	Positive
	Human	SW_human_333	*S. warneri*	cfo, pen, eri, cli	Positive
2	Dog	SE_dog_33.1	*S. epidermidis*	pen, oxa, eri	Positive
	Human	SE_human_340	*S. epidermidis*	pen, oxa, eri	Positive
3	Dog	SE_dog_83.2	*S. epidermidis*	pen, oxa	Positive
	Human	SE_human_353	*S. epidermidis*	pen, oxa	Positive
4	Dog	SS_dog_107	*S. saprophyticus*	cfo, pen	Positive
	Human	SS_human_358.2	*S. saprophyticus*	cfo, pen	Positive
5	Dog	SE_dog_316.2	*S. epidermidis*	pen, oxa	Positive
	Human	SE_human_410	*S. epidermidis*	pen, oxa	Positive
6	Dog	SH_dog_99	*S. hominis*	cfo, pen, gen, cip, eno, mbf, stx, eri	Positive
	Human	SH_human_347	*S. hominis*	cfo, pen, gen, cip, eno, mbf, stx, eri	Positive
7	Dog	SP_dog_144	*S. pseudintermedius*	pen, oxa, gen, cip, eno, mbf, stx, eri	Positive
	Human	SP_human_367	*S. pseudintermedius*	pen, oxa, gen, cip, eno, mbf, stx, eri	Positive
8	Dog	SH_dog_23	*S. haemolyticus*	cfo, pen, gen, cip, eno, mbf, stx, eri, cli	Positive
	Human	SH_human_338	*S. haemolyticus*	cfo, pen, gen, eri	Positive
9	Dog	SE_dog_216	*S. epidermidis*	pen, oxa, rif	Positive
	Human	SE_human_387	*S. epidermidis*	pen, oxa, eri	Positive
11	Dog	SA_dog_173	*S. aureus*	pen, eri	Negative
	Human	SA_human_365	*S. aureus*	pen, gen, eri,	Negative
12	Dog	SA_dog_319	*S. aureus*	pen, eri	Negative
	Human	SA_human_412	*S. aureus*	pen, eri, cli (d)	Negative
13	Dog	SA_dog_420	*S. aureus*	pen, gen, eri, cli (d)	Negative
	Human	SA_human_416	*S. aureus*	pen, eri, cli (d)	Negative
14	Dog	MS_dog_126	*M. sciuri*	pen	Positive
	Dog	MS_dog_130	*M. sciuri*	cfo, pen	Negative
	Human	MS_human_356	*M. sciuri*	susceptible to all tested antimicrobials	Negative

(cfo) Cefoxitin; (pen) Penicillin; (gen) Gentamicin; (cip) Ciprofloxacin; (eno) Enrofloxacin; (mbf) Marbofloxacin; (stx) Sulfamethoxazole; (eri) Erythromycin; (cli) Clindamycin; (rif) Rifamycin; (oxa) Oxacillin; (dox) Doxycycline; (d) Resistant with D-zone formation.

For the present study, 27 isolates were selected (14 from dogs and 13 from their respective owners), in which the same bacterial species was identified in both hosts, enabling comparative analysis between dog–owner pairs. Clinical and epidemiological information were also obtained for each dog–owner pair from the original study, including age, dog breed, occupation in the healthcare sector, previous antimicrobial use, chronic diseases, hospitalization history, hospital visits, corticosteroid use, household animal density, degree of animal confinement, and dog sleeping location. These data were used to characterize the study population and are presented in Supplementary Table S1.

### Genomic DNA extraction and quality assessment

Bacterial isolates were previously cultured on tryptic soy agar and incubated at 37 °C for 24 h under aerobic conditions. Genomic DNA extraction was performed using the Wizard® Genomic DNA Purification Kit (Promega, United States), following the manufacturer’s instructions with adaptations for the lysis of Gram-positive cocci.

Briefly, isolated colonies were inoculated into TSB broth and incubated with shaking at 37 °C for 24 h. After centrifugation, the cell pellet was subjected to enzymatic lysis using lysostaphin, followed by chemical and thermal lysis. The material was then treated with RNase, and proteins were removed by precipitation. DNA was recovered by isopropanol precipitation, washed with 70% ethanol, and subsequently rehydrated.

DNA quantification was performed by fluorometry using the Quantus Fluorometer (Promega, United States) and the QuantiFluor® ONE dsDNA system, according to the manufacturer’s recommendations. Only samples with a minimum concentration of 30 ng/μL were considered suitable for sequencing. DNA purity was assessed by spectrophotometry using a NanoDrop™ 2000 (Thermo Scientific, United States), based on A260/A280 and A260/A230 ratios. Values between 1.8–2.2 (A260/A280) and approximately 2.0 (A260/A230) were adopted as quality criteria for inclusion in subsequent analyses.

### Sequencing, genome assembly, and annotation

Whole-genome sequencing was performed using a nanopore platform (Oxford Nanopore Technologies), with the Ligation Sequencing Amplicons – Native Barcoding Kit 24 (SQK-NBD114.24), on R10.4.1 flow cells coupled to the MinION device, according to the manufacturer’s instructions (Oxford Nanopore Technologies, 2025).

Library preparation included end repair and poly-A tailing, followed by ligation of individual barcodes for sample multiplexing. After barcoding, samples were pooled, purified using magnetic beads, and subjected to ligation of sequencing adapters. The final library was loaded onto the flow cell, and sequencing was performed with real-time basecalling using the MinKNOW software.

The generated reads were initially organized and processed, with removal of adapters and barcodes using Porechop (v0.2.4). Read quality was assessed using NanoPlot (v1.41.0), considering metrics such as length distribution and average quality. *De novo* genome assembly was performed using Flye (v2.9), which is suitable for long-read data. No additional post-assembly polishing (e.g., Medaka, Racon, or Pilon) was performed, and assemblies were generated exclusively from Oxford Nanopore long-read data. Assembly quality was evaluated using QUAST (v5.0.2), based on parameters such as number of contigs, N50, and total genome size. Genome completeness and contamination were assessed using CheckM. Genome annotation was performed using Prokka (v1.14.6), which identified protein-coding genes, RNAs, and other functional elements, and standardized output files were generated for downstream analyses.

Genome annotation was performed using Prokka (v1.14.6) through the Galaxy platform with default parameters. The annotation pipeline used the reference databases integrated into Prokka to predict and annotate coding sequences, rRNAs, tRNAs, and other genomic elements. Standardized output files were generated for subsequent analyses. Sequencing statistics (total reads, mean read length, mean read quality, read N50, total bases), assembly metrics (genome size, number of contigs, assembly N50, GC content), and genome quality estimates (completeness, contamination, and sequencing coverage) for all isolates are summarized in Supplementary Table S2.

### Identification of resistance genes, virulence factors, and SCC*mec*

The detection of antimicrobial resistance determinants was performed using ResFinder v4.7.2 (Center for Genomic Epidemiology, CGE) and the Comprehensive Antibiotic Resistance Database (CARD). ResFinder analyses were conducted using the platform’s default parameters, with a minimum sequence identity threshold of 80% and a minimum alignment length of 60% for the detection of acquired antimicrobial resistance genes. CARD analyses were performed using the Resistance Gene Identifier (RGI) with the “Perfect,” “Strict,” and complete-gene criteria based on protein homology models. Identification of Staphylococcal Cassette Chromosome *mec* (SCC*mec*) elements was performed using SCC*mec*Finder v1.2 (CGE) with a sequence identity threshold of 90%, a minimum gene length coverage of 60%, and the referenced SCC*mec* database.

The identification of virulence-associated genes was performed using VirulenceFinder v2.0.5 (Center for Genomic Epidemiology, CGE). Analyses were conducted using the *Staphylococcus aureus* database, with a minimum sequence identity threshold of 90% and a minimum gene length coverage of 60%.

The similarity of resistance and virulence gene content between dog–owner pairs was estimated using the Jaccard Index (21), which is widely applied in comparative genomics studies (22, 23), based on gene presence and absence. Values were expressed as percentages and classified into three categories: high similarity (≥70%), intermediate (31–69%), and low (≤30%).

### Genetic typing (MLST)

Genetic typing of the isolates was performed in silico using Multilocus Sequence Typing (MLST), using the MLST tools from the Center for Genomic Epidemiology (CGE) and PubMLST via the Galaxy platform.

Allelic profiles were determined by analyzing species-specific housekeeping genes using standardized schemes available in the PubMLST database. The parameters used in the analyses followed the default settings of the respective tools. The seven housekeeping genes for each species are presented in Table 2.

**Table 2:** Housekeeping genes included in the multilocus sequence typing (MLST) schemes used for sequence type (ST) assignment of the bacterial species analyzed in this study.

Bacterial species	*Housekeeping* genes
*S. aureus*	*arcC, aroE, glpF, gmk, pta, tpi, yqiL*
*S. hominis*	*arcC, glpK, gtr, pta, tpiA, tuf*
*S. haemolyticus*	*arc, SH1200, hemH, leuB, SH1431, cfxE, Ribose_ABC*
*S. epidermidis*	*arcC, aroE, gtr, mutS, pyrR, tpiA, yqiL*
*S. pseudintermedius*	*ack, cpn60, fdh, purA, sar, tuf, pta*
*M. sciuri*	*ack, aroE, ftsZ, glpK, gmk, pta1, tpiA*

### Comparative genomics analyses between dog–owner pairs

Genomes from isolates obtained from dogs and their respective owners were compared to assess genetic relatedness and investigate potential intrahousehold transmission events. The analysis followed a stepwise approach, including: (i) organization and screening of assembled genomes; (ii) pairwise comparison using Average Nucleotide Identity (ANI); (iii) estimation of genomic distances in species with multiple isolates; (iv) core genome–based phylogenetic inference, when applicable; and (v) integration of results for epidemiological interpretation at the pair level.

Additionally, chromosomal architecture was evaluated through genome alignment using the MAUVE Aligner. All analyses were conducted in a Linux environment using command-line execution. Assembled genomes were organized and renamed in a standardized manner according to the epidemiological pair and host, using the convention “PairXX_dog” and “PairXX_human.” This standardization ensured greater consistency in comparative analyses, facilitating the interpretation of distance matrices and phylogenetic reconstructions.

Isolates were grouped according to previously identified bacterial species, including *Staphylococcus* spp. and *Mammaliicoccus sciuri*. This step was essential for defining analytical strategies, as core genome–based approaches require comparisons among phylogenetically related genomes.

Accordingly, two analytical scenarios were established: (i) species with multiple isolates, in which genomic distance and phylogenetic analyses could be applied; and (ii) species represented by a single pair, in which interpretation was primarily based on ANI values.

Assembled genomes were evaluated for structural quality, considering metrics such as the number of contigs, total genome size, and assembly continuity. For species with sufficient genome data, more contiguous and representative assemblies were selected as references for core-genome–based phylogenomic analyses. For species represented by a single pair, a reference genome was not required because core-genome alignment analyses were not performed.

### Genome comparison by average nucleotide identity (ANI)

Genomic relatedness between isolates within each dog–owner pair was assessed using Average Nucleotide Identity (ANI), calculated with the software fastANI (24). Comparisons were performed pairwise, considering canine and human genomes. ANI was used as a genome-wide measure of pairwise genomic relatedness, providing an estimate of the average nucleotide identity across aligned genomic fragments. In addition to ANI values, the number of aligned fragments and the total number of fragments compared were considered, allowing a joint evaluation of identity and genome coverage.

For descriptive purposes, ANI values were classified into three operational categories: (i) high similarity (≥99.7%), indicating a high degree of genomic relatedness when supported by a substantial proportion of aligned fragments; (ii) intermediate similarity (99.0–99.7%), indicating a close genetic relationship between isolates; and (iii) low similarity (<99.0%), suggesting lower genomic relatedness. These categories were used exclusively to facilitate comparative interpretation of genomic similarity and were not intended to infer direct transmission events.

The proportion of aligned fragments was used as a complementary criterion, such that high ANI values restricted to a limited fraction of the genome were interpreted with caution, as they do not necessarily reflect overall genomic similarity between isolates. Accordingly, ANI results were interpreted as indicators of genomic relatedness compatible with potential epidemiological association, rather than definitive evidence of recent transmission or clonal dissemination.

### Genomic distance analysis (mash) and population comparison

For species represented by multiple genomes (>3), an all-vs-all genomic distance analysis was performed using Mash (25), based on the MinHash approach. Unlike alignment-based methods such as ANI, Mash estimates genome-wide distances using k-mer comparisons, enabling rapid, alignment-independent evaluation of genomic relatedness. Mash was used as a complementary genome-wide metric to ANI, providing an additional assessment of genomic similarity among isolates represented by multiple genomes.

Pairwise distances were used to compare isolates within pairs (dog–owner) and across different pairs, enabling the identification of patterns of genetic relatedness and clustering within each species.

### Visualization of genomic distances

Distance matrices were processed and converted into square matrices, which were used to generate heatmaps representing genomic relatedness. These heatmaps allowed visualization of clustering and similarity patterns among isolates and were applied to species with sufficient genome numbers for comparative analysis, notably *Staphylococcus aureus* and *Staphylococcus epidermidis*.

### Core genome alignment and preparation for phylogenetic analysis

For species with multiple genomes, core-genome alignment was performed using Parsnp (21), which is well-suited for comparisons among closely related bacterial genomes. The resulting alignment was subsequently converted into a concatenated FASTA file, enabling downstream phylogenetic analyses. This process ensured the inclusion of conserved regions shared across genomes, thereby enabling more robust evolutionary inferences. Reference genomes for Parsnp analyses were selected separately for each species based on assembly quality. Assemblies with lower fragmentation (fewer contigs), genome sizes consistent with the expected species range, and greater assembly continuity were prioritized. Accordingly, Pair13_human.fasta and Pair02_human.fasta were selected as the reference genomes for *Staphylococcus aureus* and *Staphylococcus epidermidis*, respectively. The selected reference genomes were used exclusively for within-species core-genome alignment and phylogenetic reconstruction.

### Phylogenetic inference

Phylogenetic relationships among isolates were inferred from the concatenated core genome alignment using IQ-TREE 2 (26). The most appropriate evolutionary model was automatically selected using ModelFinder (27). Tree robustness was assessed using ultrafast bootstrap and SH-aLRT tests, both with 1,000 replicates, to estimate branch support and the reliability of the inferred phylogenetic relationships. No formal recombination filtering was performed prior to phylogenetic reconstruction. Therefore, the resulting phylogenetic trees were considered complementary analyses of core-genome relatedness and were not interpreted as definitive evidence of direct transmission.

### Data integration and pairwise interpretation

Results from ANI, genomic distance (Mash), and phylogenetic analyses were integrated to assess the degree of genetic relatedness between isolates from each dog–owner pair. Interpretation jointly considered: (i) high ANI values as indicators of strong genomic similarity; (ii) low Mash distances as evidence of overall genomic relatedness; and (iii) phylogenetic clustering as complementary evidence of genomic relatedness rather than proof of direct transmission.

### Comparative genomics between dog–owner pairs

Comparative genomic analyses beyond ANI were performed only for *Staphylococcus aureus* (*n* = 6) and *Staphylococcus epidermidis* (*n* = 8), as these species were represented by a sufficient number of genomes. The remaining species were evaluated exclusively using pairwise ANI analysis.

## Results

Clinical and epidemiological characteristics of the dog–owner pairs included in the study are summarized in Supplementary Table S1. Owners ranged in age from 20 to 62 years, whereas dogs ranged from 2 to 12 years of age and represented different breeds. Previous antimicrobial use within the last 6 months was reported for both dogs and owners in several households. Most dogs had no history of chronic disease, hospitalization, or recent hospital visits. Household characteristics, including the number of companion animals, degree of confinement, and dog sleeping location, varied among pairs and are provided to characterize the study population.

### Distribution of resistance genes

A total of 31 resistance genes were identified among the 27 isolates analyzed, encompassing determinants conferring resistance to different classes of antimicrobials, including *β*-lactams, aminoglycosides, macrolides, fosfomycin, chloramphenicol, and quinolones.

Among the most prevalent genes, *mecA* and *blaZ* (70.4%; 19/27) were prominent. In addition, genes associated with efflux mechanisms and disinfectant tolerance, such as *sdrM* (55.6%; 15/27) and *sepA* (48.1%; 13/27), were also frequently observed.

Within the aminoglycoside group, *aac(6′)-aph(2″)* was detected in 40.7% (11/27) of the isolates, while *ant(6)-Ia* and *aph(3′)-III* were identified in 29.6% (8/27). Genes such as *fosB* (25.9%; 7/27), *ermT*, *norC*, and *msr(A)* (22.2%; 6/27 each) showed intermediate frequencies, whereas the remaining determinants were observed at lower proportions (3.7 to 18.5%).

Overall, a high level of agreement was observed between phenotypic antimicrobial resistance and the detection of corresponding resistance determinants. The presence of *mecA* was generally associated with phenotypic resistance to oxacillin, whereas *blaZ* was frequently detected among penicillin-resistant isolates. Likewise, resistance to macrolides and lincosamides was associated with the presence of genes such as *erm(B)*, *erm(C)*, *erm(T)*, *msr(A)*, and *mph(C)*. Detailed phenotypic and genotypic resistance profiles for each isolate are presented in Supplementary Table S3.

Analysis of resistome similarity between pet–owner pairs revealed heterogeneous patterns. Pair 14 represented the only household cluster composed of three hosts (two pets and one owner). High similarity was observed in Pairs 1, 7, and 14 (100%), as well as in Pairs 2 (86.7%), 3 (85.7%), 5 (83.3%), and 4 (75%). These profiles included determinants related to *β*-lactams, aminoglycosides, fluoroquinolones, macrolides/lincosamides/streptogramins (MLS), and disinfectant resistance.

In contrast, intermediate similarity was observed in Pairs 6 (63.6%), 12 and 13 (50%), and 8 (38.7%), while Pairs 9 (27.3%) and 11 showed low similarity (25% each). The results are presented in Table 3.

**Table 3:** Distribution of antimicrobial resistance genes identified in bacterial isolates obtained from dog–owner pairs.

Pair	Host	ID sample	Bacterial species	Resistance genes	% similarity within the pair
1	Dog	SW_dog_07	*S. warneri*	*blaZ, sdrM, sepA*	100
	Human	SW_human_333	*S. warneri*	*blaZ, sdrM, sepA*	
2	Dog	SE_dog_33.1	*S. epidermidis*	*aph(3′)-III, aadD, ant(6)-Ia, mecA, blaZ, fosB, msr(A), mph(C), lnu(A), tet(K), qacJ, sepA, sdrM*	86.7
	Human	SE_human_340	*S. epidermidis*	*aph(3′)-III, aadD, ant(6)-Ia, mecA, blaZ, fosB, msr(A), mph(C), lnu(A), tet(K), qacJ, sepA, sdrM, norA, dfrC, SAT-4, ANT (4′)-Ia, dfrG*	
3	Dog	SE_dog_83.2	*S. epidermidis*	*mecA, blaZ, fosB, dfrC, sepA, sdrM*	85.7
	Human	SE_human_353	*S. epidermidis*	*mecA, blaZ, fosB, dfrC, sepA, sdrM, norA*	
4	Dog	SS_dog_107	*S. saprophyticus*	*mecA, fusD, sepA, norC*	75
	Human	SS_human_358.2	*S. saprophyticus*	*mecA, fusD, sepA*	
5	Dog	SE_dog_316.2	*S. epidermidis*	*mecA, fosB, dfrC, sdrM, norA*	83.3
	Human	SE_human_410	*S. epidermidis*	*mecA, fosB, dfrC, sdrM, norA, sepA*	
6	Dog	SH_dog_99	*S. hominis*	aac(6′)-aph(2″), ant(6)-Ia, aph(3′)-III, *mecA, blaZ, msr(A), qacJ, SAT-4, sdrM,* aph(3′)-IIIa	63.6
	Human	SH_human_347	*S. hominis*	aac(6′)-aph(2″), ant(6)-Ia, aph(3′)-III, *mecA, blaZ,nmsr(A), qacJ, str*	
7	Dog	SP_dog_144	*S. pseudintermedius*	aac(6′)-aph(2″), ant(6)-Ia, aph(3′)-III, *mecA, blaZ, erm(B), dfrG, aph(3′)-IIIa, qacG*	100
	Human	SP_human_367	*S. pseudintermedius*	aac(6′)-aph(2″), ant(6)-Ia, aph(3′)-III, *mecA, blaZ, erm(B), dfrG, aph(3′)-IIIa, qacG*	
8	Dog	SH_dog_23	*S. haemolyticus*	aac(6′)-aph(2″), ant(6)-Ia, aph(3′)-III, *mecA, blaZ, msr(A), mph(C)*	38.7
	Human	SH_human_338	*S. haemolyticus*	aac(6′)-aph(2″), ant(6)-Ia, aph(3′)-III, *mecA, blaZ, aadD, str, erm(B), erm(C), Inu(A), sepA*	
9	Dog	SE_dog_216	*S. epidermidis*	*mecA, blaZ, sdrM, sepA, aac(6′)-aph(2″)*	27.3
	Human	SE_human_387	*S. epidermidis*	*mecA, blaZ, sdrM, fosB, fusB, msr(A), mph(C), norA, norC,*	
11	Dog	SA_dog_173	*S. aureus*	*aac(6′)-aph(2″), erm(T), blaZ, norC, mgrA, S. aureus norA*	25
	Human	SA_human_365	*S. aureus*	*aac(6′)-aph(2″), erm(T), aph(2″)-Ia, str*	
12	Dog	SA_dog_319	*S. aureus*	*blaZ, erm(T), srdM, norC*	50
	Human	SA_human_412	*S. aureus*	*blaZ, erm(T), srdM, sepA, erm(C)*	
13	Dog	SA_dog_420	*S. aureus*	*blaZ, erm(T), srdM, norC, aac(6′)-aph(2″), sepA, S. aureus, norA, qacJ*	50
	Human	SA_human_416	*S. aureus*	*blaZ, erm(T), srdM, norC*	
14	Dog	MS_dog_126	*M. sciuri*	*mecA, sal(A)*	100
	Dog	MS_dog_130	*M. sciuri*	*mecA, sal(A)*	
	Human	MS_human_356	*M. sciuri*	*mecA, sal(A)*	

### Distribution of virulence genes

A total of 60 virulence-associated genes were identified among the 27 isolates analyzed, distributed across different functional categories, including adhesion, immune evasion, toxin production, extracellular enzymes, biofilm formation, secretion systems, and metabolic adaptation. The functional classification of all virulence-associated genes identified in this study is presented in Supplementary Table S4. Overall, there was a predominance of genes related to extracellular enzyme production and structural factors, whereas classical toxin and immune evasion genes exhibited a more heterogeneous distribution.

Among the most prevalent genes, *nuc* (63%; 17/27), *sspA* and *lip* (59.3% each; 16/27), were prominent, indicating a high frequency of factors associated with extracellular enzyme production. Other relevant genes included *geh* (48.1%; 13/27), *atl* and *sspB* (44.4% each; 12/27).

Genes related to adhesion and host interactions also showed notable frequencies, including *ebp* (37%; 10/27), *sdrC* and *ebh* (29.6% each; 8/27). Additionally, *clfB*, *hlb*, and *hlgC* were detected in 25.9% (7/27) of the isolates.

Genes detected at an intermediate frequency included *sdrD*, *sdrG*, *aur*, *hlgA*, and *hlgB*, each present in 22.2% (6/27) of the isolates. The immune evasion gene *scn*, the adhesin-encoding gene *sdrE*, the protease-associated gene *sspC*, and components of the ESX secretion system (*esxA*, *esaB*, *esaG*, and *essB*) were each detected in 18.5% (5/27) of the isolates. Genes associated with secretion systems, such as *essC*, as well as the adhesion-associated gene *fnbB* and the lipoprotein biosynthesis gene *lgt*, were detected in 14.8% (4/27) of the isolates were noteworthy, along with components of the biofilm operon (*icaB*. *icaC* and *icaD* 14.8%).

In contrast, several genes were observed at low frequency (≤10.3%), including *ACME*, *chp*, *coa*, *fnbA*, *eap/map*, *essA*, *hld, hly/hla, hysA, spa*, *ndk, sdrH, set5, set6* and *set26* (11.1%; 3/27). Low-frequency virulence-associated determinants included *cna*, *clfA*, *efb*, *sdrF*, *set1*, *set2*, *set3*, and *sugC*, each detected in 7.4% (2/27) of the isolates. An even lower prevalence was observed for *hpt*, *lpeA*, *icaR*, *galE*, *sbi*, *lisR*, *narX*, *sec*, *set7*, *lukD*, *lukE*, and *ybcV*, which were each identified in a single isolate (3.7%; 1/27).

The analysis of virulence gene repertoire similarity between dog–owner pairs revealed substantial variation. High similarity was observed in Pair 5 (100%), followed by Pairs 2 (90%), 1 (77.8%), and 3 (76.9%), indicating strong sharing of virulence factors between isolates. Intermediate similarity was observed in Pairs 8 (60%), 4 (44.4%), and 14 (42.8%).

In contrast, low similarity was observed in Pairs 11 (9.1%), 12 (12.5%), and 13 (12.8%), even among isolates of the same species (*Staphylococcus aureus*). Pairs 7 and 9 showed no similarity in virulence gene profiles. In Pair 7 no virulence-associated genes were detected in the *S. pseudintermedius*, using the VirulenceFinder database, preventing the identification of shared virulence determinants. In Pair 9, the canine isolate carried only the *ACME* gene, which was absent from the corresponding human isolate, resulting in no shared virulence markers between the pair. The results are presented in Table 4.

**Table 4:** Distribution of virulence-associated genes identified in bacterial isolates obtained from dog–owner pairs.

Pair	Host	ID sample	Bacterial species	Virulence genes	% similarity within the pair
1	Dog	SW_dog_07	*S. warneri*	*sdrC, sspB, sspC,* *sspA, nuc, lip, cylR2*	77,8
	Human	SW_human_333	*S. warneri*	*sdrC, sspB, sspC,* *sspA, nuc, lip, cylR2,* *sdrE, geh*	
2	Dog	SE_dog_33.1	*S. epidermidis*	*atl, ebh, clfB, ebp,* *sdrG, sspB, lip, geh,* *sspA, nuc, esaA, esaB,* *esaG, essA, essB, essC,* *esxA, hlb, sdrE*	90
	Human	SE_human_340	*S. epidermidis*	*atl, ebh, clfB, ebp,* *sdrG, sspB, lip, geh,* *sspA, nuc, esaA, esaB,* *esaG, essA, essB, essC,* *esxA, hlb, narX*	
3	Dog	SE_dog_83.2	*S. epidermidis*	*ACME, atl, ebh, sdrD, sspB,* *lip, geh, sspA,* *nuc, hlb, ebp,* *sdrH*	76,9
	Human	SE_human_353	*S. epidermidis*	*ACME, atl, ebh, sdrD, sspB,* *lip, geh, sspA, nuc,* *hlb, sdrG*	
4	Dog	SS_dog_107	*S. saprophyticus*	*atl, lip, sspA,* *nuc, sdrC, geh*	44,4
	Human	SS_human_358.2	*S. saprophyticus*	*atl, lip, sspA,* *nuc, ebp, icaA, icaB*	
5	Dog	SE_dog_316.2	*S. epidermidis*	*atl, ebh, sdrC, sdrF,* *sdrG, sdrH, sspB,* *lip, geh, sspA, nuc,* *hlb*	100
	Human	SE_human_410	*S. epidermidis*	*atl, ebh, sdrC, sdrF,* *sdrG, sdrH, sspB,* *lip, geh, sspA, nuc,* *hlb*	
6	Dog	SH_dog_99	*S. hominis*	*lip, ebp, nuc*	33,3
	Human	SH_human_347	*S. hominis*	*lip*	
7	Dog	SP_dog_144	*S. pseudintermedius*	*clfB, fnbB, icaB, sdrD,* *lip, geh, nuc, galE,* *sec, lukD, lukE, cylR2,* *lpeA, lisR, lgt*	0
	Human	SP_human_367	*S. pseudintermedius*	No hit found	
8	Dog	SH_dog_23	*S. haemolyticus*	*alt, lip, nuc*	60
	Human	SH_human_338	*S. haemolyticus*	*alt, lip, nuc,* *clfA, ebp*	
9	Dog	SE_dog_216	*S. epidermidis*	*ACME*	0
	Human	SE_human_387	*S. epidermidis*	*atl, ebh, ebp, icaA,* *icaB, icaC, icaD, icaR,* *sdrG, sspB, lip, geh,* *sspA, nuc, hlb*	
11	Dog	SA_dog_173	*S. aureus*	*hlgB, hlgC, aur, scn, atl,* *ebh, clfA, clfB, ebp,* *eap/map, efb, fnbA, fnbB,* *icaA, icaB, icaC, icaD,* *sdrC, sdrD, sdrE, spa,* *sspB, sspC, hysA, lip, geh,* *sspA, coa, nuc, chp,* *sbi, esaA, esaB, esaG,* *esxA, hly/hla, hld, set1,* *set26, set2, set3, set5,* *set6, hlgA*	9,1
	Human	SA_human_365	*S. aureus*	*hlgB, hlgC, aur, scn*	
12	Dog	SA_dog_319	*S. aureus*	*hlgA, hlgB, hlgC, aur, scn,* *cna, ebp,* *eap/map, efb, fnbA, fnbB,* *icaA, icaC, icaD,* *sdrC, sdrD, sdrE, spa,* *sspB, sspC, hysA, lip, geh,* *sspA, coa, nuc, chp,* *esaA, esaB, esaG, essB, essC,* *esxA, hly/hla, hld, set1,* *set26, set5,* *set6, set7*	12,5
	Human	SA_human_412	*S. aureus*	*hlgA, hlgB, hlgC, aur, scn*	
13	Dog	SA_dog_420	*S. aureus*	*hlgA, hlgB, hlgC, aur, scn*	12,8
	Human	SA_human_416	*S. aureus*	*hlgA, hlgB, hlgC, aur, scn,* *cna, ebp,* *eap/map, fnbA, fnbB,* *icaA, icaC, icaD,* *sdrC, sdrD, sdrE, spa,* *sspB, sspC, hysA, lip, geh,* *sspA, coa, nuc, chp,* *esaA, esaB, esaG, essB, essC,* *esxA, hly/hla, hld, set2,* *set3, set26,* *set5, set6*	
14	Dog	MS_dog_126	*M. sciuri*	*clfB, ndk, lgt, sspA, sugC*	42,8
	Dog	MS_dog_130	*M. sciuri*	*clfB, ndk, lgt*	
	Human	MS_human_356	*M. sciuri*	*clfB, ndk, lgt, sspA, sugC*	

### Characterization of *SCCmec* types and MLST profiles

A total of 27 isolates distributed across 13 dog–owner pairs were analyzed. Among *SCCmec*-positive isolates, type III was identified in Pairs 3 and 4, corresponding to 14.8% (4/27) of the isolates. Type V was detected in isolates SE_human_340, SH_human_338, and SE_human_387 (11.1%; 3/27), while type IV was observed in Pair 5 (7.4%; 2/27). Additionally, SCC*mec* elements could not be identified in 66.7% of the isolates (18/27), indicating that these strains either lacked detectable SCC*mec* structures or carried SCC*mec* elements that could not be assigned using the typing approach employed.

MLST typing analysis revealed clonal diversity among the isolates. Sequence types ST398 (*S. aureus*) and ST225 (*M sciuri)* were the most frequent, each representing 11.1% each (3/27) of the samples, including isolates SA_dog_173, SA_human_412, SA_dog_420, and Pair 14 (with 3 isolates). This was followed by ST924 (*S. epidermidis*, Pair 2), ST1067 (*S. epidermidis,* Pair 3), ST59 (*S. epidermidis,* Pair 5), and ST12 (*S. hominis*, Pair 6), each identified in two isolates (7.4%; 2/27).

Unique profiles were observed for ST3 (*S. haemolyticus*, isolate SH_dog_23), ST8 (*S. haemolyticus,* isolate SH_human_338), ST488 (*S. epidermidis*, isolate SE_dog_216), ST35 (*S. epidermidis*, isolate SE_human_387), and ST4461 (*S. aureus*, isolate SA_human_365), each corresponding to 3.7% (1/27). In addition, 14.8% (4/27) of the isolates, including Pairs 1 and 4 did not present previously described sequence types. Pair 7 and isolates SA_dog_319 and SA_human_416, were classified as non-typeable, corresponding to 17.8% (4/27).

Overall, the results indicate a predominance of isolates lacking *SCCmec*, associated with high clonal diversity. This pattern suggests the circulation of multiple bacterial lineages in the studied context, with occasional presence of clones associated with methicillin resistance. *SCCmec* and STs are summarized for the studied pairs in Table 5.

**Table 5:** Distribution of SCC*mec* types and ST identified in the isolates.

Pair	Host	Sample ID	Bacterial species	Scc*mec*	ST
1	Dog	SW_dog_07	*S. warneri*	Not detected	------
	Human	SW_human_333	*S. warneri*	Not detected	------
2	Dog	SE_dog_33.1	*S. epidermidis*	Not detected	924
	Human	SE_human_340	*S. epidermidis*	Type_V	924
3	Dog	SE_dog_83.2	*S. epidermidis*	Type_III	1,067
	Human	SE_human_353	*S. epidermidis*	Type_III	1,067
4	Dog	SS_dog_107	*S. saprophyticus*	Type_III	------
	Human	SS_human_358.2	*S. saprophyticus*	Type_III	------
5	Dog	SE_dog_316.2	*S. epidermidis*	Type_IV	59
	Human	SE_human_410	*S. epidermidis*	Type_IV	59
6	Dog	SH_dog_99	*S. hominis*	Not detected	12
	Human	SH_human_347	*S. hominis*	Not detected	12
7	Dog	SP_dog_144	*S. pseudintermedius*	Not detected	Non-typeable
	Human	SP_human_367	*S. pseudintermedius*	Not detected	Non-typeable
8	Dog	SH_dog_23	*S. haemolyticus*	Not detected	3
	Human	SH_human_338	*S. haemolyticus*	Type_V	8
9	Dog	SE_dog_216	*S. epidermidis*	Not detected	488
	Human	SE_human_387	*S. epidermidis*	Type_V	35
11	Dog	SA_dog_173	*S. aureus*	Not detected	398
	Human	SA_human_365	*S. aureus*	Not detected	4,461
12	Dog	SA_dog_319	*S. aureus*	Not detected	Non-typeable
	Human	SA_human_412	*S. aureus*	Not detected	398
13	Dog	SA_dog_420	*S. aureus*	Not detected	398
	Human	SA_human_416	*S. aureus*	Not detected	Non-typeable
14	Dog	MS_dog_126	*M. sciuri*	Not detected	225
	Dog	MS_dog_130	*M. sciuri*	Not detected	225
	Human	MS_human_356	*M. sciuri*	Not detected	225

(−---) ST is not standardized for the species.

### Comparative genomics between dog–owner pairs

Very high ANI values (≥99.75%), associated with high alignment coverage, were observed in most pairs. The highest value was identified in Pair 14 (*Mammaliicoccus sciuri*), with 99.95% identity and 914/917 aligned fragments, indicating nearly complete genome sharing between the isolates. Similar results were observed in Pair 04 (*Staphylococcus saprophyticus*) (99.86%; 895/900), as well as in Pairs 12 and 13 (*Staphylococcus aureus*) (99.79–99.82%), all with a high proportion of aligned fragments. Among *Staphylococcus epidermidis* isolates, Pairs 02, 03, and 05 showed ANI values ranging from approximately 99.75 to 99.79%, with more than 99% of fragments aligned, indicating high genomic similarity across nearly the entire genome.

Pairs 01 (*Staphylococcus warneri*) and 07 (*Staphylococcus pseudintermedius*) showed ANI values close to 99.7%, also accompanied by a high proportion of aligned fragments, suggesting relevant genomic relatedness, although slightly lower than that observed in the highest similarity group.

The lowest ANI values were observed in Pair 06 (*Staphylococcus hominis*) (99.37%; 760/766), Pair 08 (*Staphylococcus haemolyticus*) (98.71%; 781/892), and Pair 11 (*Staphylococcus aureus*) (98.7%; 684/931). In these cases, the reduction in both nucleotide identity and the proportion of aligned fragments indicates greater genomic divergence between isolates.

Overall, the results demonstrate the coexistence of pairs with high genomic similarity—consistent with potential clonal sharing—and pairs with greater divergence, suggesting multiple epidemiological scenarios within the dog–owner context.

### Genomic distance structure in *Staphylococcus aureus*

All-vs-all genomic distance analysis based on Mash revealed a heterogeneous pattern of relatedness among *S. aureus* isolates. The heatmap showed variation in intrapair similarity among the analyzed pairs (Pairs 11, 12, and 13).

The lowest distance values were observed in Pairs 12 and 13 (≈0.0010–0.0019), indicating high genomic similarity between isolates from dogs and their respective owners. These findings are consistent with the high ANI values previously observed, reinforcing the genetic relatedness within these pairs.

In contrast, Pair 11 exhibited a significantly higher distance between isolates (≈0.0147), suggesting lower genomic relatedness. This pattern was corroborated by comparisons involving the human isolate from this pair, which showed consistently higher distances relative to the other genomes (≈0.015–0.016), indicating greater genetic divergence within the analyzed dataset.

Furthermore, isolates from Pairs 12 and 13 were more closely related to each other than to those from Pair 11, forming a consistent low-distance cluster. This pattern suggests the circulation of genetically related lineages among these pairs, in contrast to the greater divergence observed in Pair 11. Overall, the genomic distance patterns observed among the analyzed isolates are summarized in Figure 1, which illustrates the distribution of Mash distances and the clustering relationships among dog–owner pairs.

**Figure 1:**
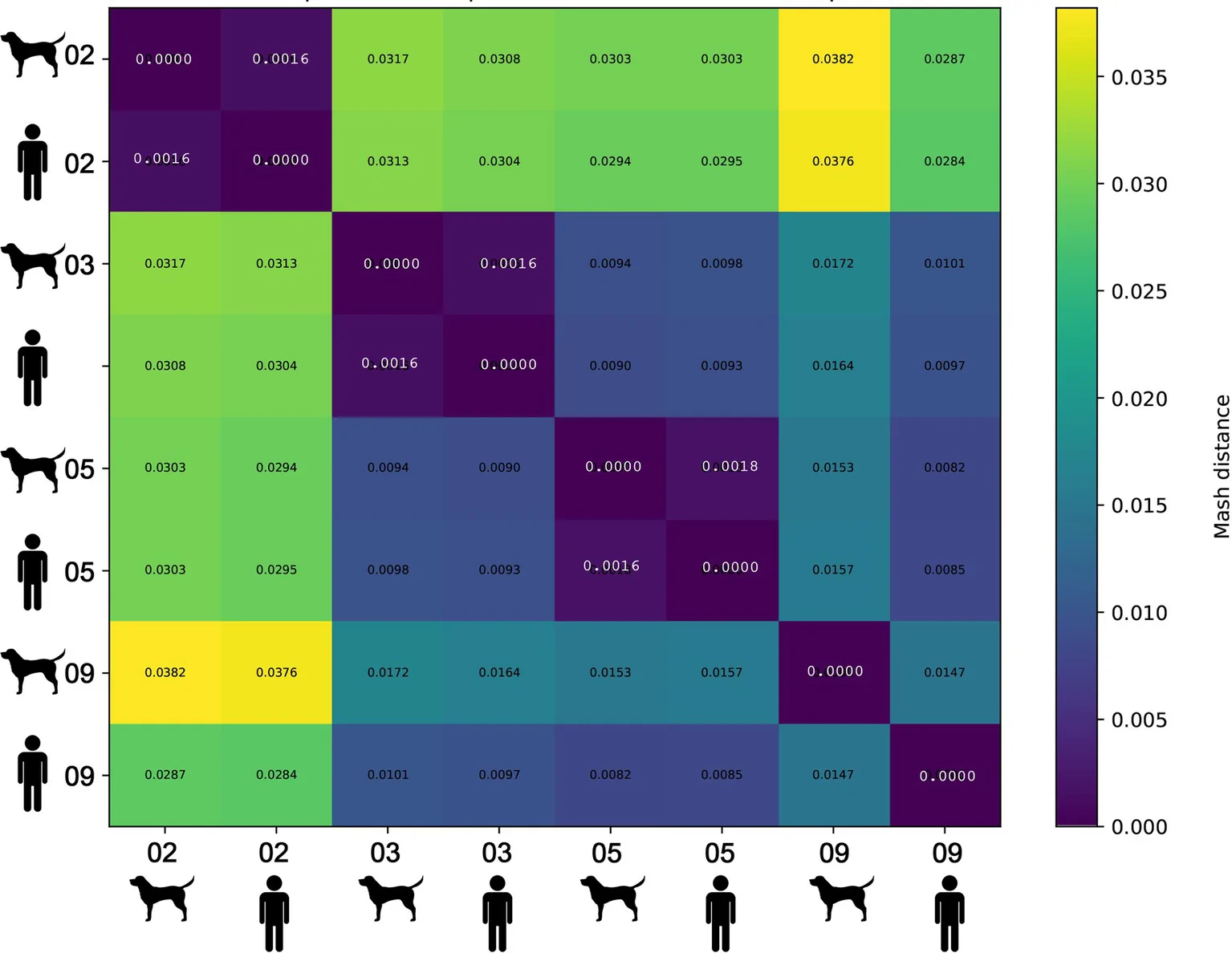
Heatmap of pairwise genomic distances (Mash distance) among *Staphylococcus epidermidis* isolates recovered from dog–owner pairs (Pairs 02, 03, 05 and 09). Each cell represents the estimated genomic distance between two genomes, with values closer to zero indicating higher genomic similarity. The color scale ranges from purple (lower genomic distance) to yellow (higher genomic distance). Rows and columns correspond to the analyzed isolates, and icons indicate the host of origin (dog or human).

### Genomic distance structure in *Staphylococcus epidermidis*

Genomic distance analysis based on Mash for *Staphylococcus epidermidis* revealed a pattern distinct from that observed in *Staphylococcus aureus*, characterized by greater organization and clearer clustering. The heatmap showed a consistent pattern of intrapair proximity among isolates from Pairs 02, 03 and 05.

In all pairs, isolates from dogs and their respective owners exhibited the lowest genomic distances (≈0.0016–0.0018), indicating high similarity and suggesting strong intrahousehold genetic relatedness.

Additionally, a subclustering structure among pairs was observed. Pair 02 showed substantially higher distances relative to Pairs 03 and 05 (≈0.029–0.031), indicating greater genetic divergence. In contrast, Pairs 03 and 05 exhibited intermediate distances between each other (≈0.009–0.010), suggesting closer relatedness and possible sharing of a more closely related lineage. The genomic relationships and clustering patterns observed among the *S. epidermidis* isolates are summarized in Figure 2, which illustrates the distribution of Mash distances and the population structure among the analyzed dog–owner pairs.

**Figure 2:**
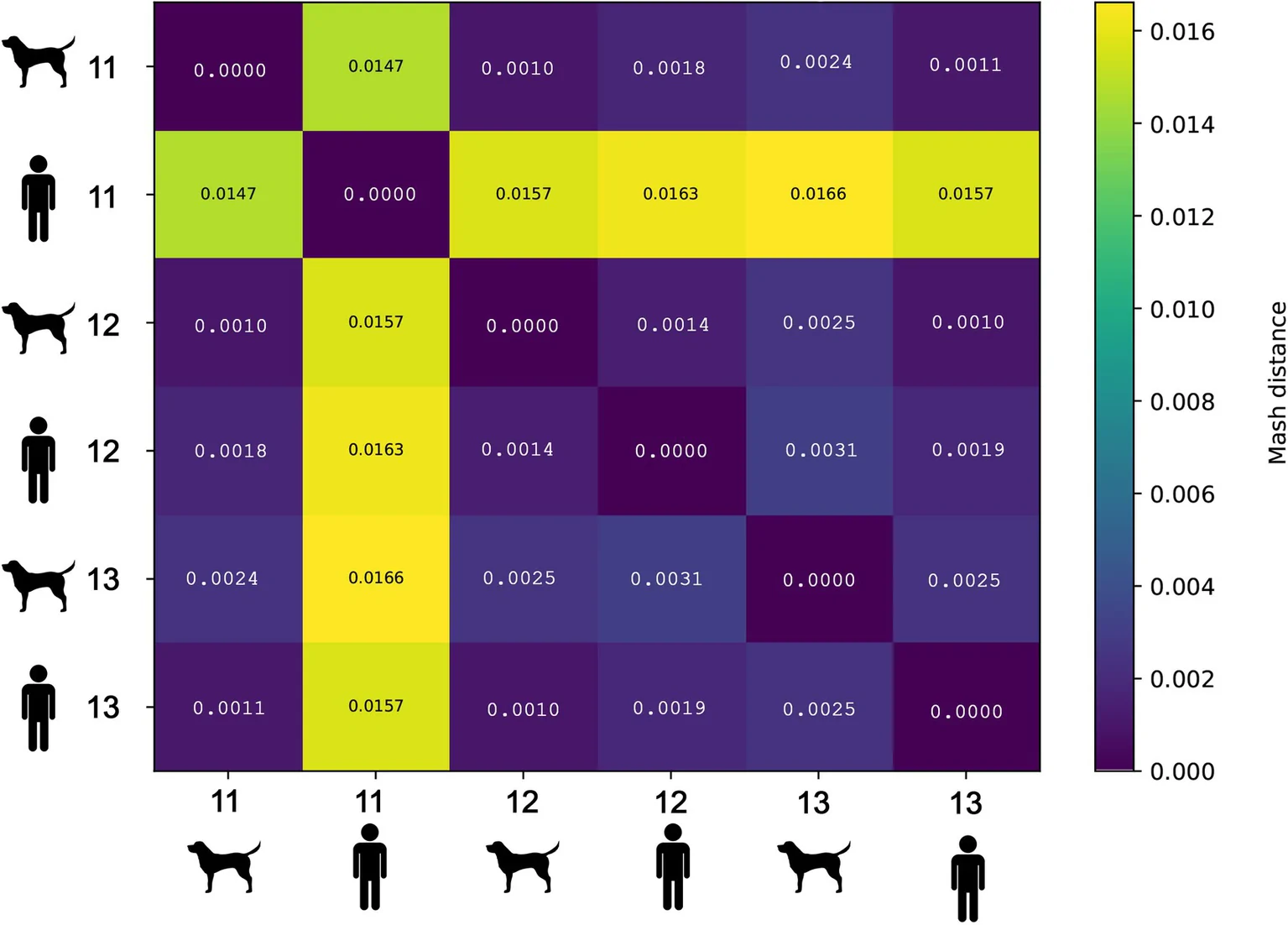
Heatmap of pairwise genomic distances (Mash distance) among *Staphylococcus aureus* isolates recovered from dog–owner pairs (Pairs 11, 12, and 13). Each cell represents the estimated genomic distance between two genomes, with values closer to zero indicating higher genomic similarity. The color scale ranges from purple (lower genomic distance) to yellow (higher genomic distance). Rows and columns correspond to the analyzed isolates, and icons indicate the host of origin (dog or human).

### Core genome phylogeny of *Staphylococcus aureus* and *Staphylococcus epidermidis*

The maximum likelihood tree of *S. aureus* (Figure 3A) revealed the absence of exclusively intrahousehold clustering among all isolates. However, Pair 12 formed a clade with maximal support, grouping both canine and human isolates, thereby reinforcing evidence of intrapair sharing. In contrast, isolates from Pair 11, although positioned within the same tree, exhibited a divergent pattern, with the human isolate showing a significantly longer branch, indicating greater evolutionary distance. This finding is consistent with ANI and Mash results, which did not support recent clonal sharing in this pair.

**Figure 3:**
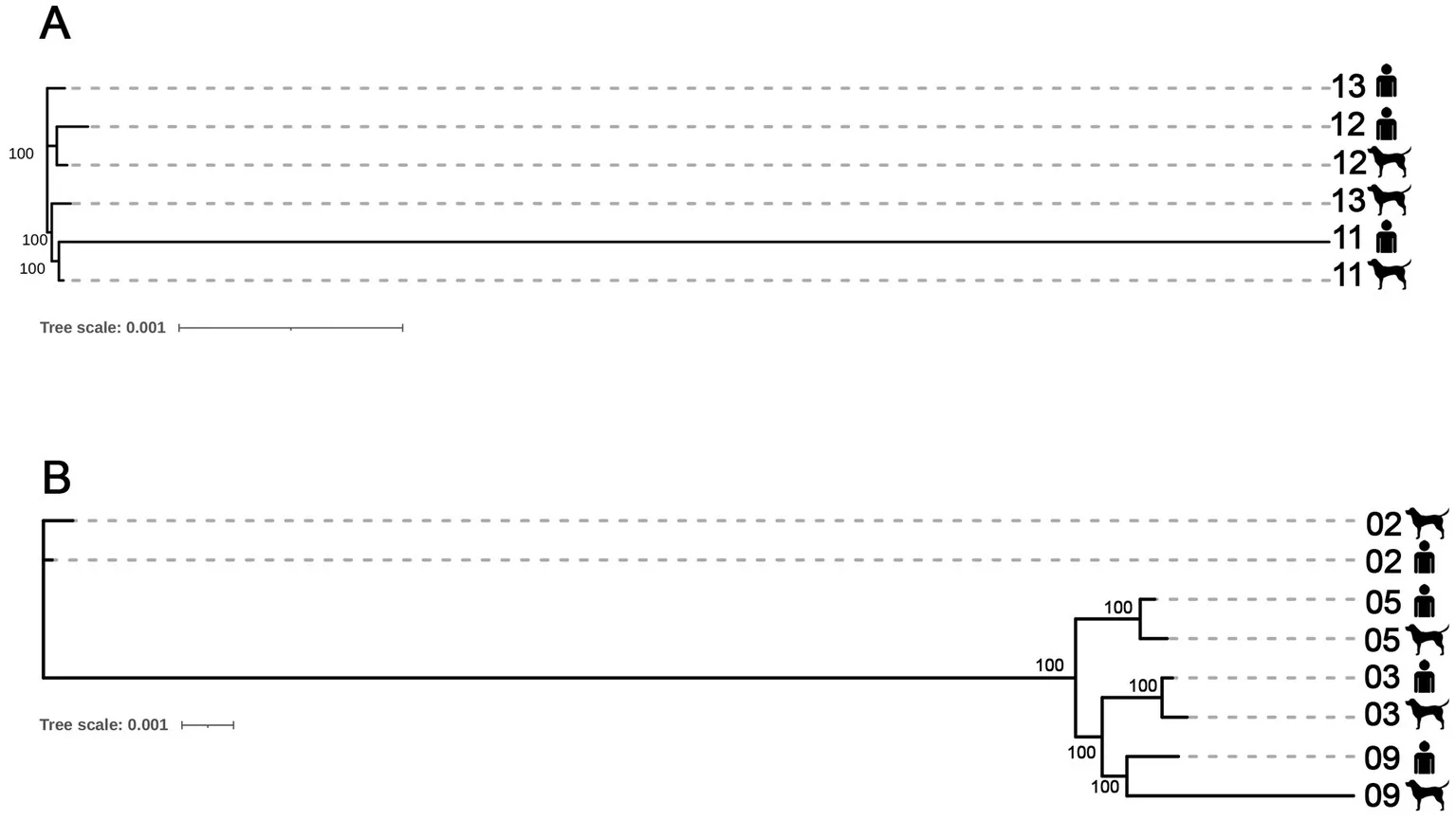
Maximum-likelihood phylogenetic trees inferred from core-genome alignments of *Staphylococcus aureus* **(A)** and *Staphylococcus epidermidis* **(B)** isolates obtained from dog–owner pairs. Phylogenetic reconstruction was performed using IQ-TREE 2. Node values represent ultrafast bootstrap support percentages (1,000 replicates), indicating the statistical support for each branch. Branch lengths are proportional to the number of substitutions per site. Pair numbers correspond to dog–owner households, and icons indicate the host of origin of each isolate (dog or human).

The canine isolate from Pair 13 clustered near other *S. aureus* isolates, suggesting high genetic relatedness; however, it was embedded within a broader lineage and did not form an exclusive clade with its corresponding pair.

For *S. epidermidis*, the phylogenetic tree (Figure 3B) showed a clearly defined pair-based structure. Isolates from Pairs 03 and 05 formed distinct clades with high statistical support, indicating strong intrapair relatedness. Pair 02, in turn, remained internally clustered but separate from the clades formed by Pairs 03 and 05, corroborating the subclustering patterns previously observed in genomic distance analyses.

Overall, the phylogenetic results reinforce the findings obtained through ANI and Mash analyses, revealing scenarios ranging from those compatible with intrahousehold clonal sharing to those indicating greater genetic divergence—particularly in *S. aureus*, whereas *S. epidermidis* exhibited a more consistent pair-based clustering structure.

### Integration of findings by pair

The integration of the different genomic approaches employed in this study—including Average Nucleotide Identity (ANI) analysis, k-mer–based genomic distances, and phylogenetic inference—allowed the identification of three distinct patterns of relationships between isolates obtained from dogs and their respective owners (Table 6).

**Table 6:** Average nucleotide identity (ANI) values and proportion of aligned genomic fragments between canine and human isolates belonging to different *Staphylococcus* spp. and *Mammaliicoccus* species.

Pair	Species	ANI (%)	Aligned fragments
Pair01	*S. warneri*	99,71	807/842
Pair02	*S. epidermidis*	99,79	833/836
Pair03	*S. epidermidis*	99,78	862/866
Pair04	*S. saprophyticus*	99,86	895/900
Pair05	*S. epidermidis*	99,75	813/819
Pair06	*S. hominis*	99,37	760/766
Pair07	*S. pseudintermedius*	99,75	827/898
Pair08	*S. haemolyticus*	98,71	781/892
Pair11	*S. aureus*	98,7	684/931
Par12	*S. aureus*	99,8	837/924
Par13	*S. aureus*	99,82	759/793
Par14	*M. sciuri* (D_1_ vs. H)	99,95	914/917
	*M. sciuri* (D_2_ vs. H)	99,85	915/916
	*M. sciuri* (D_1_ vs. D_2_)	99,85	914/917

ANI values were used as indicators of genomic relatedness and were interpreted together with genome coverage, represented by the proportion of aligned fragments.

The first pattern corresponds to pairs exhibiting a high degree of genomic relatedness, characterized by high ANI values, low genomic distances, and consistent phylogenetic clustering among isolates. This profile was identified in Pairs 02, 03, and 05, involving *Staphylococcus epidermidis*, and in Pair 12, involving *Staphylococcus aureus*, indicating highly similar genomes and a close genetic relationship between canine and human isolates.

A second group included pairs showing moderate to high genomic relatedness but without clear exclusivity within the household. In these cases, although high levels of genomic identity were observed, the isolates were embedded within broader lineages or the comparative analysis was limited by the reduced number of available genomes. This pattern was observed in Pairs 01 (*Staphylococcus warneri*), 04 (*Staphylococcus saprophyticus*), 07 (*Staphylococcus pseudintermedius*), 13 (*Staphylococcus aureus*), and 14 (*Mammaliicoccus sciuri*), suggesting genetic similarity that may reflect either epidemiological relatedness within households or the broader circulation of closely related lineages.

Finally, a third pattern was identified, characterized by lower ANI values and greater overall genomic divergence between canine and human isolates. This profile was observed in Pairs 06 (*Staphylococcus hominis*), 08 (*Staphylococcus haemolyticus*), and 11 (*Staphylococcus aureus*), with the latter showing particularly pronounced discrepancies between human and canine isolates, consistent with distinct genetic backgrounds. The integrated interpretation of the genomic relationships identified among the analyzed dog–owner pairs is summarized in Table 7.

**Table 7:** Integrated interpretation of genomic relationships among bacterial isolates obtained from dog–owner pairs based on average nucleotide identity (ANI), mash genomic distances, and phylogenetic inference.

Pair	Species	ANI (%)	Mash (distance)	Phylogeny	Integrated interpretation
Pair01	*S. warneri*	99,71	Low	ND	Moderately related isolates (limited comparative context)
Pair02	*S. epidermidis*	99,79	Very low	Cluster intrapair	Highly related isolates
Pair03	*S. epidermidis*	99,78	Very low	Cluster intrapair	Highly related isolates
Pair04	*S. saprophyticus*	99,86	ND	ND	Moderately related isolates (limited comparative context)
Pair05	*S. epidermidis*	99,75	Very low	Cluster intrapair	Highly related isolates
Pair06	*S. hominis*	99,37	ND	ND	Genetically distinct isolates
Pair07	*S. pseudintermedius*	99,75	Low	ND	Moderately related isolates (limited comparative context)
Pair08	*S. haemolyticus*	98,71	ND	ND	Genetically distinct isolates
Pair11	*S. aureus*	98,70	High	Divergent	Genetically distinct isolates
Pair12	*S. aureus*	99,80	Very low	Intrapair cluster	Highly related isolates
Pair13	*S. aureus*	99,82	Very low	Intrapair cluster in a broad lineage	Moderately related isolates within broader lineages
Pair14	*M. sciuri*	99,85–99,95	ND	ND	Moderately related isolates (limited comparative context)

ND (not defined): analysis not performed for the corresponding species (fewer than 3 genomes available).

The table summarizes the degree of genomic relatedness observed for each pair and the corresponding interpretation of genomic similarity patterns identified through the combined analytical approaches.

## Discussion

The results of this study reveal a relevant scenario of antimicrobial resistance among *Staphylococcus* spp. and *Mammaliicoccus sciuri* isolates obtained from dogs and their respective owners, characterized by a high frequency of resistance genes, including *mecA* and *blaZ*, as well as determinants conferring resistance to multiple classes of antimicrobials. This resistome profile reflects a concerning level of multidrug resistance within the household environment, highlighting the potential role of companion animals as reservoirs and amplifiers of antimicrobial resistance, as previously demonstrated (10, 28), although shared resistance patterns alone are not sufficient to confirm direct transmission events.

Similarly, (29) reported the presence of multidrug-resistant *M. sciuri* in critical veterinary environments, reinforcing the role as a reservoir of resistance genes and its zoonotic potential. Together, these studies support the notion that both coagulase-negative staphylococci and *M. sciuri* contribute to the maintenance and dissemination of resistance determinants across different ecological niches. However, international evidence indicates that the presence of resistance genes does not necessarily imply direct transmission between hosts. Studies conducted in different geographical contexts (30) demonstrated that although resistant species are frequently found in companion animals, genetic correspondence with human isolates is not always observed, leading to the interpretation that multiple acquisition sources are involved. This highlights the importance of integrating high-resolution genomic approaches to disentangle shared exposure from true transmission events.

The overall agreement observed between phenotypic antimicrobial resistance and the detection of corresponding resistance genes supports the usefulness of whole-genome sequencing for predicting antimicrobial resistance profiles in staphylococci. In particular, the frequent detection of *mecA* and *blaZ* was consistent with resistance to oxacillin and penicillin, respectively, whereas resistance to macrolides and lincosamides was commonly associated with the presence of *erm*, *msr*, and *mph* genes. Similar genotype–phenotype correlations have been widely reported in staphylococci from both human and animal sources, especially for β-lactam and macrolide resistance determinants (31, 32). Nevertheless, previous studies have also demonstrated that the relationship between resistance genotype and phenotype is not always absolute, since resistance expression may be influenced by gene regulation, mutations affecting gene functionality, differences in gene expression levels, and limitations inherent to resistance gene databases (31, 32). Therefore, genomic and phenotypic approaches should be regarded as complementary tools for the comprehensive characterization of antimicrobial resistance.

Regarding virulence factors, there was a predominance of genes involved in extracellular enzyme production, such as *nuc*, *sspA*, and *lip*, corroborating studies highlighting the role of these molecules in immune evasion and host adaptation (33, 34). These factors may enhance colonization capacity and persistence on host surfaces, potentially facilitating inter-host transmission under conditions of close contact. The high frequency of these genes has also been reported in coagulase-negative staphylococci of clinical origin (35), although their distribution may vary according to ecological niche, as observed in environmental isolates (36). In this context, the detection of similar virulence profiles in isolates from dogs and owners further supports the hypothesis of shared microbial pools within households, even in the absence of confirmed clonal identity. It is important to note that the virulence gene analysis was performed using the *S. aureus* database available in VirulenceFinder. Therefore, isolates belonging to other staphylococcal species, particularly *S. pseudintermedius*, may have had species-specific virulence determinants that were not represented in the reference database. This limitation may have resulted in an underestimation of the virulence gene repertoire in some isolates and should be considered when interpreting pairwise similarity values.

The presence of adhesion-related genes such as *ebp*, *sdrC*, and *ebh*, reinforces the importance of host–pathogen interaction in colonization, a key step for the establishment of infection. These adhesion factors play a critical role in mediating attachment to host tissues and abiotic surfaces, facilitating stable colonization in both human and animal hosts (37). These findings are consistent with the literature, which describes surface proteins as central determinants of staphylococcal pathogenesis (32), and they are frequently identified in studies of transmission between humans and animals (38). In the context of close human–animal contact, such as that observed in household environments, these molecules may contribute to the successful establishment and maintenance of shared bacterial populations. On the other hand, the variability observed in the distribution of these genes among isolates has also been reported (39), suggesting the influence of ecological factors and bacterial species, as well as potential differences in host adaptation and niche specialization.

Genes associated with biofilm formation, particularly the *ica* operon, showed intermediate frequency, consistent with studies indicating that this mechanism is essential for persistence, especially in *S. epidermidis* (35, 40). Biofilm formation enhances bacterial survival under adverse conditions, including host immune responses and antimicrobial exposure, and is considered a key factor in chronic colonization (41). However, their lower frequency relative to hospital-derived isolates suggests that the household environment may exert weaker selective pressure on the maintenance of this phenotype, potentially reflecting differences in antimicrobial exposure, host density, and environmental stability between clinical and domestic settings.

The heterogeneous distribution of toxin genes, including hemolysins and enterotoxins, is consistent with previous reports showing that coagulase-negative staphylococci possess a more limited toxigenic repertoire than *Staphylococcus aureus* (33, 35). This finding suggests a distinct virulence strategy in these species, characterized by a lower reliance on acute toxin-mediated damage and a greater association with mechanisms of colonization, persistence, and host adaptation. In the analyzed isolates, virulence appears to be more strongly linked to the ability to establish long-term host interactions and persist in shared environments, potentially increasing opportunities for inter-host transmission, than to toxin production alone. This methodological aspect may also explain the absence of detected virulence genes in the *S. pseudintermedius* isolate from Pair 7, which resulted in an apparent 0% similarity in virulence gene repertoire. Rather than indicating a true lack of virulence-associated determinants, this finding likely reflects the limited ability of an *S. aureus*-based database to detect virulence genes specific to other staphylococcal species.

Characterization of SCC*mec* elements revealed a predominance of isolates lacking detectable SCC*mec* cassettes, along with the identification of SCC*mec* types III and V. However, it is important to consider that the SCC*mec* typing tool used in this study is primarily based on *Staphylococcus aureus* reference sequences, which may limit its applicability to *Mammaliicoccus* species and other non-aureus staphylococci. Therefore, the absence of SCC*mec* identification does not necessarily indicate the true absence of this genetic element, as divergent, incomplete, or previously uncharacterized SCC*mec* structures may not be recognized by the current database. Consequently, interpretations regarding the dissemination and genetic context of methicillin resistance should be made with caution. This limitation should be considered when evaluating SCC*mec*-negative or non-typeable isolates and highlights the need for complementary genomic approaches to further characterize these elements. This pattern suggests a heterogeneous distribution of methicillin resistance mechanisms, potentially reflecting differences in selective pressures and ecological niches between isolates. These results are consistent with studies demonstrating broad SCC*mec* diversity in animal-derived isolates, often associated with lineages adapted to multiple hosts (42, 43). MLST analysis revealed clonal diversity, with emphasis on ST398, widely described as an animal-associated clone with recognized zoonotic potential (44, 45). The presence of multiple sequence types, as well as non-typeable isolates, suggests the circulation of still poorly characterized lineages, particularly in community settings, highlighting the potential for underrecognized reservoirs of genetic diversity and antimicrobial resistance.

Comparative analysis of dog–owner pairs revealed both high genetic similarity and significant divergence. The presence of pairs with high genetic correspondence supports the potential interspecies strain sharing, as described in previous studies (38, 46). However, the coexistence of pairs with low similarity indicates that sharing is not universal and may be influenced by environmental, behavioral, and antimicrobial exposure factors, suggesting that multiple transmission pathways and independent acquisition events coexist within the same setting.

Clinical and epidemiological data collected from the participating households provide additional context for the genomic findings. Several dog–owner pairs shared characteristics that have previously been associated with *Staphylococcus* carriage and interspecies bacterial exchange, including close human–animal contact, indoor housing, and bed sharing, whereas previous antimicrobial exposure was reported for both dogs and owners in some households. Exposure to healthcare settings was also identified among a subset of participants, either through healthcare-related occupations or hospital visits. *Staphylococcus aureus* colonization may occur concurrently in dogs and their owners (47), whereas the epidemiology of staphylococci at the human–animal interface is multifactorial, involving host characteristics, antimicrobial exposure, and environmental interactions rather than a single transmission route (48). In the present study, similar epidemiological characteristics were observed among pairs displaying both high and low genomic relatedness, reinforcing that bacterial sharing at the human–animal interface is likely driven by a complex interplay of host, bacterial, behavioral, and environmental factors rather than by any single epidemiological variable.

Comparative genomics results further support this interpretation. High ANI values (>99.7%) associated with high genomic coverage are widely recognized as indicative of clonal relatedness (24), supporting the hypothesis of recent bacterial sharing or very close genomic relatedness in some pairs.

In contrast, intermediate or slightly lower ANI values likely reflect ongoing genomic divergence, which may arise from microevolution following transmission or from acquisition of closely related but distinct strains. These patterns highlight the dynamic nature of bacterial populations at the human–animal interface, where both recent transmission and long-term evolutionary processes may occur simultaneously. Genomic distance and phylogenetic analyses revealed distinct patterns between species, with *S. aureus* showing greater heterogeneity and *S. epidermidis* exhibiting more structured clonal patterns, consistent with studies describing differences in population dynamics between these species (35). This distinction may reflect differences in ecological versatility and transmission dynamics, with *S. epidermidis* behaving as a more stable commensal lineage and *S. aureus* exhibiting higher adaptive flexibility and diversification potential.

Integration of the data allowed the identification of three genomic relationship patterns: highly related isolates, moderately related isolates, and genetically distinct isolates. These patterns represent a continuum of genomic relatedness rather than discrete categories, reflecting varying degrees of similarity between canine and human isolates. This pattern reflects the complexity of staphylococcal epidemiology, which involves not only direct transmission but also circulation across multiple reservoirs, including the environment and the community (38, 39). Such complexity underscores the importance of considering both host-associated and external sources when interpreting genomic similarity patterns in household settings.

Overall, the findings of this study reinforce that *Staphylococcus* spp. and *Mammaliicoccus sciuri* act as important reservoirs of antimicrobial resistance in the household context, but that transmission dynamics are heterogeneous and multifactorial. Rather than supporting a single epidemiological pattern, our results suggest the coexistence of highly related strains, genetically distinct lineages, and possible epidemiological connections between humans and companion animals. These results highlight the importance of genomic surveillance and support the adoption of integrated One Health–based strategies at the human–animal interface, including rational antimicrobial use, hygiene measures, and continuous monitoring of bacterial populations in humans and companion animals.

This study demonstrates that the genomic relationships among *Staphylococcus* spp. and *Mammaliicoccus sciuri* isolates at the human–animal interface are heterogeneous and best understood as a continuum, encompassing highly related strains, genetically distinct lineages, and varying degrees of genomic similarity. By integrating comparative genomics with resistome and virulome profiling, our findings reinforce the role of companion animals as potential reservoirs of antimicrobial-resistant bacteria within household environments. These results underscore the need to incorporate pets into genomic surveillance frameworks and reinforce the importance of One Health–based strategies to better understand the genomic diversity, antimicrobial resistance profiles, and epidemiological relationships of bacterial populations at the human–animal interface.

This study has some limitations that should be considered when interpreting the results. First, the sample size was relatively small and restricted to a limited number of dog–owner pairs, which may limit the generalizability of the findings. Second, the cross-sectional design provided only a single time-point assessment, preventing evaluation of temporal dynamics and precluding determination of the direction or timing of bacterial acquisition. Third, environmental sampling was not performed. Consequently, potential environmental reservoirs within households could not be assessed, and the contribution of environmental sources to the observed genomic relationships remains unknown.

Although clinical and epidemiological information was collected for all participating dog–owner pairs, the limited sample size precluded formal assessment of associations between epidemiological variables and genomic relatedness. Therefore, these data were used only to characterize the study population and provide contextual support for the genomic findings.

Comparative genomic analyses provided valuable insights into genomic relatedness among isolates; however, SNP-based phylogenetic analyses, pairwise SNP distance estimation, and recombination filtering were not performed. Consequently, genomic similarity and phylogenetic clustering should not be interpreted as definitive evidence of direct or recent transmission but rather as indicators of genomic relatedness compatible with possible intrahousehold clonal sharing.

Additionally, genome assemblies were generated exclusively from Oxford Nanopore long-read sequencing data. Although this approach enables highly contiguous genome assemblies, residual base-calling errors may persist and could affect fine-scale comparative analyses. Furthermore, plasmid content and the genomic context of mobile genetic elements, including prophages, insertion sequences, and integrons, were not specifically investigated, limiting the assessment of the mechanisms underlying antimicrobial resistance dissemination.

Future studies incorporating larger sample sizes, longitudinal sampling, environmental isolates, short-read validation or hybrid assemblies, SNP-based comparative genomics with recombination filtering, and comprehensive characterization of mobile genetic elements will be important to further elucidate bacterial ecology and antimicrobial resistance at the human–animal interface.

## Data Availability

The raw sequencing reads generated in this study have been deposited in the NCBI Sequence Read Archive (SRA) under BioProject accession number PRJNA1480536. Individual isolate metadata are available in the BioSample database under accession numbers SAMN61033061–SAMN61033086. Genome assemblies will be made available through GenBank upon completion of the submission process.
