# Supplementary material for: Comparative genomic analysis of Staphylococcus spp. and Mammaliicoccus sciuri suggests potential sharing between dogs and their owners
Source: Front Public Health. 2026 Oct 2;14:1879619. doi: 10.3389/fpubh.2026.1879619 (PMC13672652; doi:10.3389/fpubh.2026.1879619)
Supplement: Supplementary file 1 [file Data_Sheet_1.pdf]

Supplementary Table S1. Clinical and epidemiological characteristics of the dog–owner pairs included in this study.

Pair	Dog ID	Owner ID	Dog Breed	Age (y)	Neutered	Antimicrobial use†	Chronic disease	Hospitalization†	Bath location	Owner Age (y)	Healthcare worker	Antimicrobial use†	Chronic disease	Hospital visit†	Corticosteroid use† (Dog/Owner)	Household Confinement ‡	Animals in household	Dog sleeping location
1	SW_dog_07	SW_human_333	Mixed breed	8	Yes	Cephalexin (2 years ago)	No	No	Home	20	Yes	Azithromycin (2 months ago)	No	No	No / Yes	Indoor	6	Owner's bed
2	SE_dog_33.1	SE_human_340	Poodle	12	Yes	Yes	Ehrlichiosis / Cardiac disease	No	Home	53	No	No	No	Yes	Yes / Yes	Semi-confined	NR	Owner's bed
3	SE_dog_83.2	SE_human_353	Mixed breed	10	Yes	Yes (2×)	No	No	Home	23	Yes	No	No	Yes	No / No	Indoor	2 dogs, 2 cats	Backyard
4	SS_dog_107.1	SS_human_358.2	Golden Retriever	5	No	No	No	No	Backyard	35	Yes	No	No	Yes (3×)	No / No	Indoor	4 dogs, 2 cats	Veranda
5	SE_dog_316.2	SE_human_410	Mixed breed	5	No	Yes (1×)	No	No	Home	20	Yes	Yes	No	Yes (1×)	No / No	Indoor	3 dogs	Kennel
6	SH_dog_99	SH_human_347	Pinscher	8	Yes	Yes (3×)	No	No	Home	62	No	Yes	No	Yes (3×)	No / Yes	Indoor	4 dogs, 4 cats	Owner's bed

7	SP_dog_144	SP_human_367	Mixed breed	10	Yes	Yes	No	No	Home	49	No	No	No	No	No / No	Semi-confined	30 animals	Owner's bed
8	SH_dog_23	SH_human_338	Mixed breed	5	Yes	Yes (>1 year ago)	No	No	Home	20	Yes	No	No	Yes (>6 months)	Yes / No	Semi-confined	10 animals	Backyard
9	SE_dog_216	SE_human_387	Mixed breed	2	Yes	Yes (2×)	No	No	Pet shop	55	No	No	Hashimoto disease	Yes (1×)	No / No	Indoor	5 dogs, 3 cats	Bedroom
11	SA_dog_173	SA_human_365	Mixed breed	4	Yes	Yes (1×)	No	No	Home	44	No	Yes	No	No	No / No	Indoor	NR	Bedroom
12	SA_dog_319	SA_human_412	Labrador Retriever	9	No	Enrofloxacin	Articular disease	No	Home	58	No	No	Articular disease	No	No / No	Semi-confined	10 animals	Backyard
13	SA_dog_420	SA_human_416	Cocker Spaniel	8	No	No	Articular disease	No	Pet shop	21	Yes	No	No	Yes	No / No	Indoor	1 dog	Owner's bed
14	MS_dog_130	MS_human_356	Mixed breed	6	Yes	Yes (2×)	No	No	Home	38	No	Yes	Bronchitis	No	No / No	Indoor	1 dog	Bedroom
	MS_dog_126		Mixed breed	6	Yes	Yes (2×)	No	No	Home						No / No	Indoor	1 dog	Bedroom

Supplementary Table S2 - Sequencing, assembly, and genome quality metrics of *Staphylococcus* and *Mammaliicoccus* isolates sequenced using Oxford Nanopore Technology (ONT). Read statistics, including total reads, mean read length, mean read quality, read N50, and total bases, were obtained using NanoStat. Genome assembly metrics, including genome size, number of contigs, assembly N50, and GC content, were generated with QUAST. Genome completeness and contamination estimates were assessed using CheckM. Sequencing coverage was calculated as the ratio between the total number of sequenced bases and the assembled genome size.

Pairs	Identity	Species	Total Reads	Mean Read Length (bp)	Mean Read Quality (Q)	Read N50 (bp)	Total Bases (Mb)	Coverage (×)	Genome Size (bp)	No. Contigs	Assembly N50 (bp)	GC (%)	Completeness (%)	Contamination (%)
1	SW_dog_07	*S. warneri*	4,385	8,181.5	10.4	17,363	35.9	14.2	2,534,582	4	1,979,594	32.8	88.2	1.2
	SW_human_333	*S. warneri*	17,608	7,569.3	9.8	10,684	133.3	55.0	2,421,904	1	2,421,904	32.7	94.2	0.2
2	SE_dog_33.1	*S. epidermidis*	18,003	4,075.5	10.1	9,678	73.4	29.2	2,511,594	2	2,508,675	32.2	92.6	0.2
	SE_human_340	*S. epidermidis*	52,014	3,382.5	10.2	5,716	175.9	70.2	2,506,050	1	2,506,050	32.2	93.6	0.3
3	SE_dog_83.2	*S. epidermidis*	8,013	8,151.8	9.7	13,714	65.3	25.1	2,605,844	5	2,529,025	32.1	91.0	0.6
	SE_hum	*S. epidermidis*	19,393	8,628.7	9.7	12,370	167.3	64.6	2,592,095	4	2,528,878	32.1	92.9	0.3

	an_353													
4	SS_dog_107	*S. saprophyticus*	12,153	9,002.6	9.8	11,976	109.4	40.5	2,702,798	1	2,702,798	33.2	93.4	1.7
	SS_human_358.2	*S. saprophyticus*	8,014	7,791.6	9.7	13,958	62.4	23.0	2,716,247	2	2,702,360	33.1	92.8	1.1
5	SE_dog_316.2	*S. epidermidis*	36,047	9,010.1	9.7	12,290	324.8	132.0	2,460,576	2	2,441,414	32.2	92.8	1.0
	SE_human_410	*S. epidermidis*	4,003	11,342.2	9.8	19,003	45.4	18.5	2,451,690	2	2,442,118	32.2	88.1	0.3
6	SH_dog_99	*S. hominis*	33,168	2,451.5	10.1	3,648	81.3	35.3	2,303,551	4	2,274,736	31.6	92.8	0.1
	SH_human_347	*S. hominis*	7,267	3,953.4	10.0	13,035	28.7	12.3	2,335,842	7	2,276,871	31.7	74.4	1.9
7	SP_dog_144	*S. pseudintermedius*	167,925	2,371.4	10.0	4,069	398.2	147.5	2,700,179	6	2,655,893	37.4	70.8	0.4
	SP_human_367	*S. pseudintermedius*	81,715	753.5	9.9	1,165	61.6	22.8	2,699,117	75	98,584	37.3	89.5	0.8
8	SH_dog_23	*S. haemolyticus*	7,994	8,232.4	9.5	16,957	65.8	24.6	2,678,959	3	2,622,394	32.8	86.3	0.4
	SH_	*S.*	64,105	5,145.2	9.6	6,793	329.8	128.8	2,560,654	5	2,517,893	32.8	93.6	0.2

	human_338	*haemolyticus*												
9	SE_dog_216	*S. epidermidis*	2,428	8,689.4	10.2	12,551	21.1	8.2	2,568,228	4	1,190,735	—	70.8	0.4
	SE_human_387	*S. epidermidis*	12,021	9,572.5	9.8	17,191	115.1	46.1	2,496,636	4	2,418,976	32.1	97.0	0.2
11	SA_dog_173	*S. aureus*	48,024	5,193.0	10.3	10,239	249.4	89.1	2,797,614	4	2,784,732	32.9	97.5	0.1
	SA_human_365	*S. aureus*	11,992	1,895.5	10.1	3,088	22.7	9.1	2,490,650	185	19,679	33.2	57.2	2.0
12	SA_dog_319	*S. aureus*	32,019	8,357.0	9.8	15,949	267.6	96.5	2,773,165	1	2,773,165	33.0	94.5	0.2
	SA_human_412	*S. aureus*	304,916	524.7	10.0	661	160.0	58.5	2,734,279	119	46,475	32.9	93.6	1.3
13	SA_dog_420	*S. aureus*	92,074	4,798.6	9.8	8,207	441.8	161.1	2,743,030	1	2,743,030	32.9	95.3	1.9
	SA_human_416	*S. aureus*	131,862	869.7	10.2	1,165	114.7	41.0	2,796,676	256	17,619	32.8	92.4	3.2
14	MS_dog_126	*M. sciuri*	87,945	1,915.3	10.1	3,764	168.4	61.2	2,753,566	2	2,749,436	32.6	97.5	2.5
	MS	*M. sciuri*	10,715	5,086.7	9.6	11,04	54.5	19.8	2,750,705	2	2,748,216	32.6	93.9	2.7

	_dog_130					6								
	MS_human_356	*M. sciuri*	47,931	2,266.2	10.2	3,692	108.6	39.5	2,753,057	2	2,749,522	32.6	99.3	2.4

Supplementary Table S3. Phenotypic antimicrobial resistance profiles and corresponding resistance genes detected by whole-genome sequencing in staphylococcal isolates recovered from pets and owners. Antimicrobial susceptibility profiles were compared with the presence of acquired resistance determinants and resistance-associated genes identified through genomic analysis.

Pair	Host	Species	Phenotypic resistance profile	Resistance genes detected
1	Dog	*S. warneri*	CFO, PEN, ERY, CLI	*blaZ, sdrM, sepA*
	Owner	*S. warneri*	CFO, PEN, ERY, CLI	*blaZ, sdrM, sepA*
2	Dog	*S. epidermidis*	PEN, OXA, ERY	*aph(3')-III, aadD, ant(6)-Ia, mecA, blaZ, fosB, msr(A), mph(C), lnu(A), tet(K), qacJ, sepA, sdrM*
	Owner	*S. epidermidis*	PEN, OXA, ERY	*aph(3')-III, aadD, ant(6)-Ia, mecA, blaZ, fosB, msr(A), mph(C), lnu(A), tet(K), qacJ, sepA, sdrM, norA, dfrC, SAT-4, ANT(4')-Ia, dfrG*
3	Dog	*S. epidermidis*	PEN, OXA	*mecA, blaZ, fosB, dfrC, sepA, sdrM*
	Owner	*S. epidermidis*	PEN, OXA	*mecA, blaZ, fosB, dfrC, sepA, sdrM, norA*
4	Dog	*S. saprophyticus*	CFO, PEN	*mecA, fusD, sepA, norC*

	Owner	*S. saprophyticus*	CFO, PEN	*mecA, fusD, sepA*
5	Dog	*S. epidermidis*	PEN, OXA	*mecA, fosB, dfrC, sdrM, norA*
	Owner	*S. epidermidis*	PEN, OXA	*mecA, fosB, dfrC, sdrM, norA, sepA*
6	Dog	*S. hominis*	CFO, PEN, GEN, CIP, ENO, MBF, STX, ERY	*aac(6')-aph(2''), ant(6)-Ia, aph(3')-III, mecA, blaZ, msr(A), qacJ, SAT-4, sdrM, aph(3')-IIIa*
	Owner	*S. hominis*	CFO, PEN, GEN, CIP, ENO, MBF, STX, ERY	*aac(6')-aph(2''), ant(6)-Ia, aph(3')-III, mecA, blaZ, msr(A), qacJ, str*
7	Dog	*S. pseudintermedius*	PEN, OXA, GEN, CIP, ENO, MBF, STX, ERY	*aac(6')-aph(2''), ant(6)-Ia, aph(3')-III, mecA, blaZ, erm(B), dfrG, aph(3')-IIIa, qacG*
	Owner	*S. pseudintermedius*	PEN, OXA, GEN, CIP, ENO, MBF, STX, ERY	*aac(6')-aph(2''), ant(6)-Ia, aph(3')-III, mecA, blaZ, erm(B), dfrG, aph(3')-IIIa, qacG*
8	Dog	*S. haemolyticus*	CFO, PEN, GEN, CIP, ENO, MBF, STX, ERY, CLI	*aac(6')-aph(2''), ant(6)-Ia, aph(3')-III, mecA, blaZ, msr(A), mph(C)*
	Owner	*S. haemolyticus*	CFO, PEN, GEN, ERY	*aac(6')-aph(2''), ant(6)-Ia, aph(3')-III, mecA, blaZ, aadD, str, erm(B), erm(C), lnu(A), sepA*
9	Dog	*S. epidermidis*	PEN, OXA, RIF	*mecA, blaZ, sdrM, sepA, aac(6')-aph(2'')*

	Owner	*S. epidermidis*	PEN, OXA, ERY	*mecA, blaZ, fosB, fusB, msr(A), mph(C), norA, norC, sdrM*
11	Dog	*S. aureus*	PEN, ERY	*aac(6')-aph(2''), erm(T), blaZ, norC, mgrA, norA*
	Owner	*S. aureus*	PEN, GEN, ERY	*aac(6')-aph(2''), erm(T), aph(2'')-Ia, str*
12	Dog	*S. aureus*	PEN, ERY	*blaZ, erm(T), sdrM, norC*
	Owner	*S. aureus*	PEN, ERY, CLI (D-test)	*blaZ, erm(T), sdrM, sepA, erm(C)*
13	Dog	*S. aureus*	PEN, GEN, ERY, CLI (D-test)	*blaZ, erm(T), sdrM, norC, aac(6')-aph(2''), sepA, norA, qacJ*
	Owner	*S. aureus*	PEN, ERY, CLI (D-test)	*blaZ, erm(T), sdrM, norC*
14	Dog	*Mammaliicoccus sciuri*	PEN	*mecA, sal(A)*
	Dog	*Mammaliicoccus sciuri*	CFO, PEN	*mecA, sal(A)*
	Owner	*Mammaliicoccus sciuri*	PEN	*mecA, sal(A)*

Supplementary Table S4 - Functional classification of virulence-associated genes identified among the staphylococcal isolates. Genes were categorized according to their reported biological roles, including adhesion, biofilm formation, immune evasion, toxin production, secretion systems, proteolytic activity, metabolism/adaptation, and regulatory functions.

Gene	Encoded product / Main function	Functional category
nuc	Thermonuclease	Invasion/Dissemination
sspA	V8 serine protease	Protease
lip	Lipase	Extracellular enzyme
atl	Major autolysin involved in initial attachment and biofilm formation	Adhesion/Biofilm
sspB	Cysteine protease (staphopain B)	Protease
ebp	Elastin-binding protein	Adhesion
geh	Glycerol ester hydrolase (lipase)	Extracellular enzyme
ebh	Extracellular matrix-binding protein homolog	Adhesion
sdrC	Serine-aspartate repeat protein C	Adhesion
clfB	Clumping factor B	Adhesion

hlb	β-hemolysin	Toxin
hlgC	Gamma-hemolysin component C	Toxin
aur	Aureolysin (metalloprotease)	Protease
hlgA	Gamma-hemolysin component A	Toxin
hlgB	Gamma-hemolysin component B	Toxin
icaA	Polysaccharide intercellular adhesin (PIA) synthesis	Biofilm
sdrD	Serine-aspartate repeat protein D	Adhesion
sdrG	Fibrinogen-binding protein	Adhesion
esaA	Type VII secretion system structural component	Type VII secretion system (T7SS)
esaB	Type VII secretion system regulator	Type VII secretion system (T7SS)
esaG	Type VII secretion-associated protein	Type VII secretion system (T7SS)
essB	Type VII secretion system component	Type VII secretion system (T7SS)

icaC	PIA export and synthesis	Biofilm
scn	Staphylococcal complement inhibitor	Immune evasion
sdrE	Adhesin associated with factor H binding	Adhesion/Immune evasion
sspC	Protein associated with the Ssp protease system	Protease
essC	ATPase of the Type VII secretion system	Type VII secretion system (T7SS)
esxA	Secreted effector of the Type VII secretion system	Type VII secretion system (T7SS)
fnbB	Fibronectin-binding protein B	Adhesion
icaB	PIA deacetylase	Biofilm
icaD	Biofilm formation protein	Biofilm
lgt	Lipoprotein diacylglyceryl transferase	Lipoprotein maturation
chp	Chemotaxis inhibitory protein	Immune evasion
clfA	Clumping factor A	Adhesion

coa	Coagulase	Immune evasion
eap/ map	Extracellular adherence protein / MHC class II analog protein	Adhesion/Immune evasion
essA	Type VII secretion system component	Type VII secretion system (T7SS)
fnbA	Fibronectin-binding protein A	Adhesion
hld	δ-hemolysin	Toxin
hla	α-hemolysin	Toxin
hysA	Hyaluronidase	Invasion enzyme
ndk	Nucleoside diphosphate kinase	Adaptation/Survival
sdrH	Serine-aspartate repeat protein H	Adhesion
set5	Staphylococcal enterotoxin-like protein 5	Toxin
set6	Staphylococcal enterotoxin-like protein 6	Toxin
set26	Staphylococcal enterotoxin-like protein 26	Toxin

spa	Protein A	Immune evasion
ACME	Arginine catabolic mobile element	Colonization/Adaptation
cna	Collagen adhesin	Adhesion
efb	Extracellular fibrinogen-binding protein	Immune evasion
sdrF	Serine-aspartate repeat protein F	Adhesion
set.1	Staphylococcal enterotoxin-like protein 1	Toxin
set.2	Staphylococcal enterotoxin-like protein 2	Toxin
set.3	Staphylococcal enterotoxin-like protein 3	Toxin
sugC	ABC carbohydrate transporter ATP-binding protein	Metabolism/Adaptation
galE	UDP-galactose 4-epimerase	Metabolism/Cell wall biosynthesis
hpt	Hexose phosphate transporter	Metabolism/Adaptation
lpeA	Adhesion-associated lipoprotein	Adhesion

lukD	Leukocidin D component	Toxin
lukE	Leukocidin E component	Toxin
narX	Nitrate sensor histidine kinase	Regulation
sbi	Staphylococcal binder of immunoglobulin	Immune evasion
sec	Staphylococcal enterotoxin C	Superantigen/Toxin
set.7	Staphylococcal enterotoxin-like protein 7	Toxin
icaR	Transcriptional repressor of the *ica* operon	Biofilm regulation
lisR	Response regulator of the LisRK two-component system	Regulation
ybcV	Putative stress adaptation-associated protein	Other/Adaptation
